# Effects of oily fish intake on cardiovascular risk markers, cognitive function, and behavior in school-aged children: study protocol for a randomized controlled trial

**DOI:** 10.1186/s13063-016-1647-z

**Published:** 2016-10-21

**Authors:** Camilla T. Damsgaard, Lotte Lauritzen, Hanne Hauger, Stine Vuholm, Marie N. Teisen, Christian Ritz, Max Hansen, Janni Niclasen, Christian Mølgaard

**Affiliations:** 1 Department of Nutrition, Exercise and Sports, Faculty of Science, University of Copenhagen, Rolighedsvej 26, 1958 Frederiksberg C, Denmark; 2 Division of Risk Assessment and Nutrition, National Food Institute, Technical University of Denmark, Søborg, Denmark; 3 Department of Psychology, Faculty of Social Science, University of Copenhagen, Copenhagen, Denmark; 4 Centre for Collaborative Health, Aarhus University, Aarhus C, Denmark

**Keywords:** n-3 PUFA, Child health, Dietary recommendations, Cognitive function, Cardiovascular risk factors, Mental health

## Abstract

**Background:**

Most children in Western populations do not meet recommendations for fish consumption. Oily fish is an important source of n-3 long-chain polyunsaturated fatty acids (LCPUFA), which reduce blood pressure and plasma triacylglycerol in adults and may affect cognitive development and behavior. However, to our knowledge, the potential effects of oily fish on cardiometabolic health, cognitive function, and behavior in children have not been investigated. The aim of the FiSK Junior study is to investigate the effects of oily fish consumption on cardiovascular risk markers, cognitive function, and behavior in healthy children.

**Methods/design:**

We are conducting a randomized controlled trial with 8- to 9-year-old Danish children, comparing the effect of consuming 300 g/week of oily fish with poultry (control) for 12 weeks between August 2016 and June 2017. The primary outcomes are blood pressure and fasting plasma triacylglycerol, which will be measured at baseline and endpoint. In addition, we will assess erythrocyte fatty acid composition (compliance), heart rate, plasma cholesterol, markers of glucose homeostasis, growth and body composition, dietary intake, and physical activity and sleep. We will also examine effects on cognitive function (attention, memory, and executive functions) by using standardized tests, behavior and emotions by administering parent-rated questionnaires and child interviews, and we will measure physiological stress response and cortisol levels. We need 150 children to complete the trial to detect a between-groups difference of 2.7 mmHg in diastolic blood pressure and 0.13 mmol/L in plasma triacylglycerol; thus, we aim to recruit 200 children. All outcomes will be analyzed in completer analysis supplemented with sensitivity analyses for the primary outcomes, and attention will be given to potential sex and genotype specificity.

**Discussion:**

The results of the FiSK Junior study are expected to fill important gaps in the current knowledge about the importance of dietary fish and n-3 LCPUFA for children’s health and development, and may be used when setting dietary recommendations.

**Trial registration:**

ClinicalTrials.gov identifier: NCT02809508. Registered on 22 June 2016.

**Electronic supplementary material:**

The online version of this article (doi:10.1186/s13063-016-1647-z) contains supplementary material, which is available to authorized users.

## Background

Fish is an important dietary source of iodine, selenium, zinc, and high-quality proteins. Oily fish is also the main dietary source of n-3 long-chain polyunsaturated fatty acids (LCPUFA), which have been shown to provide protection from cardiovascular disease [1] and may affect cognitive function and behavior. Most Western countries recommend two to four servings of fish weekly, typically half thereof as oily fish, but especially children do not meet these recommendations [2–4]. Danes are recommended to consume 350 g/week of fish, whereof 200 g/week should be oily fish such as salmon, mackerel, and herring [5], but less than 10 % of Danish children aged 4 to 9 years reach this intake [3]. Oily fish is also an important natural source of vitamin D, of which most Danes consume too little [3]. Vitamin D deficiency is common at northern latitudes during winter, when the contribution of vitamin D from sun exposure is negligible [6]. Low vitamin D status can impair children’s bone health [7] and may also affect immune function and cardiovascular health [8].

Fish consumption is associated with reduced cardiovascular mortality in adults [9]. This is presumed to be due mainly to the content of n-3 LCPUFA, as these fatty acids (typically administered as fish oil) are well known to reduce cardiovascular risk factors, especially blood pressure [10], heart rate [11], and plasma triacylglycerol [12] in adults. Our previous randomized trials with fish oil provided to infants and adolescents indicated that n-3 LCPUFA have comparable effects in children [13–15]. Randomized controlled trials of oily fish in adults rather than fish oils have shown mixed results, but most indicate that consumption of oily fish lowers blood pressure and plasma triacylglycerol (see, e.g., [16, 17]). To our knowledge, no randomized trial has investigated the effect of fish consumption per se on cardiovascular health in children.

During brain growth, n-3 LCPUFA, specifically docosahexaenoic acid (DHA, 22:6-n-3), are incorporated at uniquely high levels in the brain [18], which may confer increased needs for these fatty acids in childhood. The rate of incorporation has been shown to be diet-dependent and is believed to play a role in the cognitive development of the child [18]. Most trials investigating the effects of n-3 LCPUFA on cognitive function have been conducted with formula-fed infants or with infants of mothers receiving fish oil. Meta-analyses of these studies have not shown pronounced benefits of n-3 LCPUFA on neurodevelopmental outcomes during the first 2 years of life [19]. However, this may be because the effect of n-3 LCPUFA is sex-dependent [20, 21]. Few studies of young children have examined the effects on specific cognitive functions, and with conflicting results, possibly due to methodological limitations [22]. In addition to cognitive function, studies investigating the effects of n-3 LCPUFA on behavior and emotional outcomes are sparse. A few randomized trials in healthy schoolchildren have shown that children supplemented with fish oil had reduced impulsivity, antisocial behavior, and physical activity during school hours [23–25], but this needs more investigation.

Most studies of fish and n-3 LCPUFA have been conducted with males or without specific attention to sex. However, recent work indicates that some effects of n-3 LCPUFA in children may be sex-specific, especially in relation to blood pressure and cognitive function [26, 27]. Moreover, studies indicate that the effects of n-3 LCPUFA, and therefore potentially also oily fish intake, are genotype-dependent [28].

### Aim

In the FiSK (Fish, children, health, and cognition (Fisk, børn, Sundhed og Kognition)) Junior study, we will investigate whether intake of oily fish compared with poultry affects cardiovascular risk markers, primarily blood pressure and plasma triacylglycerol, in children. Moreover, we will assess the effects on cognitive function, behavior, and emotions and give special attention to potential sex and genotype specificity. This paper presents the rationale, design, and potential implications of the study.

## Methods

### Study design and ethics

**Fig. 1:**
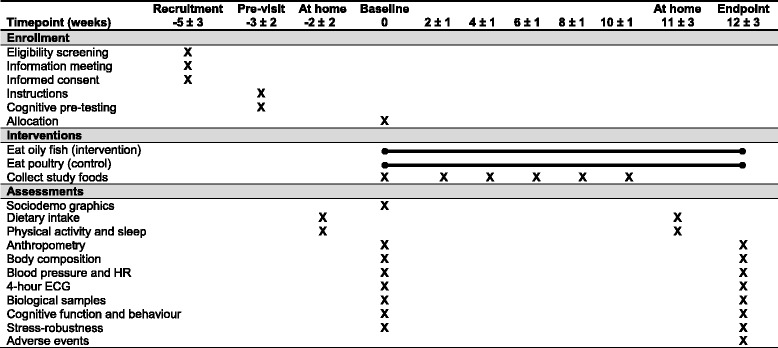
Overview of study schedule and main activities. *ECG* Electrocardiogram, *HR* Heart rate

The FiSK Junior study is a randomized controlled parallel trial in which Danish children will receive 300 g/week of oily fish or poultry (control) for 12 ± 3 weeks between August 2016 and June 2017. Measurements and biological sampling will be performed at baseline and endpoint at the Department of Nutrition, Exercise and Sports (NEXS), University of Copenhagen, Frederiksberg, Denmark (Fig. 1). See Additional file 1 for an overview of the Standard Protocol Items: Recommendations for Interventional Trials (SPIRIT) 2013 checklist items.

The study protocol has been approved by the Committee on Biomedical Research Ethics for the Capital Region of Denmark (H-16018225) and was registered with ClinicalTrials.gov (NCT02809508) on 22 June 2016. The study will be conducted in accordance with the guidelines in the Declaration of Helsinki, and informed written consent will be obtained from all custody holders of the children. Also, we will follow the Danish Act on Processing of Personal Data for all data collected in the study.

### Participants

Participants will be healthy 8- to 9-year-old children living in the Capital Region of Denmark. Children must speak Danish, like oily fish and chicken, not consume oily fish more than once per week, and must not take any n-3 LCPUFA supplements 3 months prior to the intervention start. Moreover, parents must read and speak Danish in order to be properly informed about the study procedures, and the household should not contain more than five people. Exclusion criteria for the children are serious chronic illnesses and diseases that may interfere with the study outcomes, diagnosed attention-deficit/hyperactivity disorders or other psychiatric illnesses, medication that may affect study outcomes, and concomitant participation in other studies involving dietary supplements or blood sampling. In addition, only one child from each household can participate in the study.

Children of the relevant age and residence will be identified through the Danish Civil Registration System and will be recruited by invitation letter to the parents. Participants may also be recruited through advertisements in Danish newspapers, through networks and social media such as Facebook, and by notices in schools. Parents who express interest will be invited to an informational meeting together with their child, after which they can decide if they wish to participate. If a child is withdrawn from the study, we will ask the parents about the reason for withdrawal. Responding is voluntary, and we will not put pressure on the parents to make them respond.

### Randomization, allocation, concealment, and blinding

Randomization will be performed in blocks of 12 children to ensure approximately equal allocation to the two study groups over the year. A staff member not involved in data collection will produce a computer-generated randomization list with 200 consecutive numbers, each linked to one of the two intervention groups, from which we will produce 200 sealed, sequentially numbered envelopes containing the randomly assigned allocation for that number. The randomization list will be generated from a prespecified seed using the extension package “blockrand” for R version 3.3.0 [29]. The order in which participants finish the baseline visit will determine their number in the randomization list (and thereby which envelope and allocation they receive). By this procedure, participants and investigators will be blinded to the allocation until the baseline measurement has been completed. However, owing to the nature of the intervention foods, blinding during the intervention is not possible.

### Dietary intervention

#### Study foods and instructions

The fish group will receive oily fish (>6 g fat/100 g) primarily as fresh or frozen fillets of Aquaculture Stewardship Council-certified farmed salmon fillets, but also as cold lunch products such as salmon fish cakes, canned mackerel, marinated herring, smoked trout, and salmon sausages. In order to provide comparable treatments, the control group will receive different cuts of frozen, organic chicken (minced, whole, breast, and thighs) and chicken lunch products such as liver pate, chicken sausages, and chicken meatballs. The participants will be asked to consume 300 g/week of the intervention products, operationalized as two times per week for dinner and three times per week for lunch. Parents will be asked to substitute some of the fish, poultry, and meat usually consumed by the child with the study products and apart from this to maintain the child’s usual dietary and physical activity habits during the intervention.

Parents will collect study foods free of charge at NEXS every 1–2 weeks. Fish and poultry for dinner will be provided for the whole family/household, whereas lunch products will be provided for the child only. Parents will also receive a booklet containing inspiration and easy recipes for preparation of study foods and side dishes. Recipes will be matched between the chicken and fish groups with regard to type of dish and energy and fat content.

#### Safety of study foods

We have thoroughly considered the safety aspects of the study foods. Two groups of unwanted substances are of special interest in relation to fish intake: methylmercury as well as dioxins and dioxin-like polychlorinated biphenyls (DDPCB). Assuming a weekly intake of 200 g of farmed salmon, 80 g of mackerel, and 20 g of herring, the study fish will provide 8.3 μg of methylmercury and 208 pg of DDPCB per week. The tolerable weekly intake (TWI) for these substances (i.e., the estimated amount that can be ingested weekly over a lifetime without appreciable health risk) is 1.3 μg/kg [30] and 14 pg toxic equivalents (TEQ)/kg body weight, respectively [31]. This corresponds to 36 μg of methylmercury and 385 pg TEQ DDPCB per week in an average Danish 8-year-old weighing 27.5 kg. Hence, it is estimated that the study fish will provide 23 % and 54 % of the children’s TWI for methylmercury and DDPCB, respectively. As fish is the main source of methylmercury in the Danish population [32], it is considered very unlikely that any of the participants will have an intake of methylmercury above the TWI. However, fish contributes only about 30 % of the intake of dioxins and PCB, whereas 70 % is from nonfish sources such as milk, cheese, meat, and fats [32]. The total mean intake of DDPCB among Danish children has been estimated to 0.87 pg/day per kilogram of body weight [32]. An average Danish 8-year-old child is therefore expected to have a DDPCB intake from nonfish sources of 0.61 pg/day per kilogram of body weight, or 118 pg/week. In the general population, there is wide variation in the exposure to DDPCB, caused mainly by the large variation in fish consumption [32]. However, because the families are provided with appropriate amounts of fish products and instructions to meet the intake of 300 g/week and are not expected to eat markedly more than that, we expect less variation and that their estimated total intake of DDPCB from study fish and nonfish sources will be around 208 + 118 = 326 pg TEQ per week, which is well below the TWI. Also, the families are instructed to replace some of the meat in their diet with the study fish, and therefore the actual mean intake of DDPCB is likely to be lower than calculated. Even though there are individual variations in the intake of DDPCB from sources other than the intervention diet, it is considered unlikely that the children’s intake of DDPCB during the intervention will exceed the TWI. The study is thus considered safe.

### Measurements

#### Primary outcomes

Although we are interested to see if oily fish consumption can improve children’s overall cardiovascular risk profile, the primary outcomes of the study will be fasting venous plasma triacylglycerol and resting diastolic blood pressure measured in the morning before blood sampling. Local anesthetic patches will be provided to the child to relieve the discomfort of blood sampling. Resting blood pressure will be measured in triplicate by using an automated device with the child lying down after a 10-minute rest, and the mean of the last two measurements will be calculated.

#### Secondary outcomes

##### Cardiovascular health

Because the pre-blood sample situation may be stressful for the participants, we will also measure blood pressure during the 3 h after blood sampling at up to two additional occasions, each time in triplicate and after a 10-minute rest. This step is used to explore whether potential effects of the intervention manifest differently under relaxed and stressful conditions and to link the cardiovascular and behavioral outcomes. Also, diastolic measurements will be supported by systolic blood pressure and heart rate measurements. In addition, children’s heart rhythm will be assessed by continuous recording of electrocardiograms using a Holter recording device throughout the 4-h visit at baseline and endpoint. The recordings will be analyzed with respect to heart rate and heart rate variability by Jeppe Hagstrup Christensen, MD, a clinical professor in the Department of Clinical Medicine, Aalborg University Hospital, Aalborg, Denmark. Other secondary outcomes are plasma total cholesterol, low-density lipoprotein cholesterol, high-density lipoprotein cholesterol, insulin, glucose, glycated hemoglobin, C-reactive protein, and adipokines.

##### Cognitive function

Cognitive function will be measured after consumption of a standardized breakfast using the following computer-based tests from the Cambridge Neuropsychological Test Automated Battery (CANTAB) [33]: the Motor Screening Test, Reaction Time, Rapid Visual Information Processing, Spatial Working Memory, and Paired Associates Learning. Further, the children will perform the d2 Test of Attention in Danish [34], a modified Flanker test [35], and the Stroop test [36] in a Danish version. These are all internationally recognized, validated, standardized tests that assess specific areas of cognitive function with a focus on attention, memory, and executive functions. The tests will be performed by trained project personnel supervised by a psychologist, and to avoid interference by learning effects, the children will pretest the CANTAB tests and the d2 Test of Attention prior to the baseline examination. The cognitive measurements will be supported by analysis of brain-derived neurotrophic factor (BDNF) in the blood samples. BDNF plays an important role in neuronal survival, and in rodents DHA has been shown to affect BDNF concentrations in brain areas important for attention, executive functions, and memory [37].

##### Stress robustness

At baseline and endpoint, children’s physiological stress response will be assessed using a cold pressor task where the child submerges his or her dominant hand in a tub containing cold water for a maximum of 1 minute. We will follow the guidelines of von Baeyer et al. [38] for the use of this task in children, but with the water temperature modified to 5 ± 2 °C. Children will be instructed that they can remove their hand from the water if they find the test too uncomfortable and will be reminded about this during the test if they display discomfort. Immediately before and after the test, we will measure the child’s blood pressure and heart rate, and before and 20 minutes after the test, we will collect a 0.5- to 2-ml saliva sample to be analyzed for cortisol concentration. Together with the heart rhythm recorded continuously during the test, these outcomes will be used to assess the child’s acute physiological stress response. Finally, a small tuft of hair (approximately 5–20 mg) will be cut from the posterior vertex of the child’s head. The 3 cm of hair located most closely to the scalp will be analyzed for cortisol concentrations, reflecting cortisol and hence stress levels over the last 3 months [39].

##### Growth and body composition

Height, weight, and waist circumference at the level of the umbilicus will be measured by standard procedures, and body mass index z-scores will be calculated. Body composition will be measured by bioimpedance with the child resting in lying position and by triplicate measurements of triceps and subscapularis skinfold thicknesses using a skinfold caliper, and fat mass index and lean mass index will be calculated. We previously found effects of fish oil on the insulin-like growth factor (IGF) axis [40], which stimulates bone and body growth in children, and therefore we will also measure IGF-1, its binding protein, and potentially other growth and bone markers in the blood samples at baseline and endpoint.

#### Other outcomes

##### Behavior

Child behavioral and emotional outcomes will be measured by questionnaires to be filled in by the parents, specifically the Strengths and Difficulties Questionnaire [41], the Behavior Rating Inventory of Executive Function questionnaire [42], and the KINDL® quality of life questionnaire developed for healthy children [43]. Parents will receive a short oral instruction in this by trained project personnel supervised by a psychologist. The child version of the KINDL questionnaire will also be administered by interview with the children. 

##### Physical activity and sleep

Children’s physical activity and sleep will be recorded by using triaxial accelerometers (ActiGraph, Pensacola, FL, USA) during 7 consecutive days and 8 nights prior to the baseline and endpoint visits. Parents will record the times of the child’s awakening and falling asleep every day during the recording, and these recordings will be used to define sleep and wake time. Physical activity and sleep will both be seen as outcomes because these behavior-related measures may be affected by the intervention, but they may also be used as covariates because they may affect the primary outcomes.

##### Compliance

Compliance with the oily fish will be assessed as n-3 LCPUFA in erythrocytes, which reflects intake during the last 3–4 months. Also, parents will be asked to record the child’s consumption of study fish and poultry on precoded sheets every week during the intervention period and to hand them in when collecting new study foods.

##### Nutrient biomarkers

We will measure serum 25-hydroxyvitamin D at baseline and endpoint because high consumption of oily fish may increase the vitamin D status of the participants. We will also measure serum hemoglobin and ferritin to assess whether the intervention affects iron status (e.g., if the study fish and chicken provided replaces red meat in the diet).

##### Genetics

To assess potential effect modification by genotype, we will analyze polymorphisms in genes coding for proteins involved in the metabolism and signaling of polyunsaturated fatty acids such as the fatty acid desaturase genes, peroxisome proliferator-activated receptor genes, and genes encoding cyclooxygenases [28].

#### Additional data

##### Dietary intake

Children’s dietary intake will be recorded prior to the baseline and endpoint visits. The parents will be required to fill in dietary records of the child’s diet for 4 consecutive days, covering 3 weekdays and 1 weekend day, using Internet-based software available for personal computer and smartphone use [44]. Household measures will be used when weighting of the foods is not possible. Additionally, parents will be given a food frequency questionnaire to assess the child’s intake of fish, poultry, and other meats.

##### Background information

At baseline, sociodemographic information will be collected and pubertal stage will be evaluated by the child, if necessary aided by a parent, using a self-administered questionnaire on breast development and age at menarche for girls and development of pubic hair for boys. Questionnaires on sun behavior will be administered at both baseline and endpoint.

##### Adverse events

Although we do not expect any adverse events related to the study to occur, parents will be asked to contact us if they experience any adverse events or reactions in their children during the study. At the endpoint visit, we will specifically ask whether they have experienced any adverse events. All reported adverse events will be recorded.

### Sample size calculation

Power calculations were performed using data from a large sample of Danish 8- to 11-year-olds [45]. In order to detect a difference of less than 0.5 SD of the outcomes i.e., 2.7 mmHg in diastolic blood pressure and 0.13 mmol/L in triacylglycerol between the fish and poultry groups with a significance level of α=0.05 and 80 % power, a total of 150 children will need to complete the study. The differences correspond to 4 % and 20 % reductions in blood pressure and triacylglycerol, respectively, which are considered relevant and obtainable. In order to allow for a 15–20 % dropout rate and insufficient blood sampling, we aim to recruit 200 children.

### Data analysis considerations

Outcomes will be analyzed in linear models with adjustment for baseline values, and the difference between the fish and poultry groups will be estimated. All data will be analyzed as completer’s analysis (i.e., including only participants who have available data from both baseline and endpoint for a given outcome). For the primary outcomes and associated cardiovascular risk markers, we will also conduct sensitivity analyses. These will include intention-to-treat analysis of all subjects randomized, with imputation of missing values using the baseline observation carried forward method and, if needed, also imputation of missing baseline values. For outcomes where differences are found, we will conduct supportive linear regression analyses exploring potential dose-response associations between erythrocyte n-3 LCPUFA content and outcomes. Secondarily, we will evaluate sex × treatment interactions. We will also investigate potential effect modification by genotype.

With an increased intake of oily fish or poultry, there is a risk that some children will gain more weight and body fat than expected during normal growth. This may be unevenly distributed between the groups and may impact the cardiovascular outcomes. In that case, we will adjust the outcomes for body mass index or fat mass index to separate the effects of the diets and weight gains. Analysis along the same line of thinking will be performed in case of other unanticipated differences occurring between the two groups.

## Discussion

In most Western countries, official dietary recommendations advocate increased consumption of fish for both children and adults. Recommendations for children are based on the high content of important nutrients in fish, as well as extrapolations from studies in adults where fish and n-3 LCPUFA consumption have been shown to be cardioprotective. However, to underpin such recommendations and get more insight into the public health and biological potential of fish consumption, high-quality trials conducted in children are needed. To our knowledge, the FiSK Junior study will be the first randomized controlled trial to investigate the impact of oily fish intake on cardiovascular risk markers in healthy children. Although very few children get lifestyle diseases, elevated cardiovascular risk markers and the metabolic syndrome are seen already in childhood [46]. The Bogalusa Heart Study [47] and the Cardiovascular Risk in Young Finns Study [48] have demonstrated that blood lipid profile and blood pressure show tracking from childhood and adolescence to adulthood. Therefore, small but persistent reductions in risk markers in childhood are likely to be important for long-term cardiovascular risk.

To our knowledge, the FiSK Junior study is also the first randomized trial to investigate effects of oily fish intake on cognitive function and behavior in children attending primary school in a high-income country. The study will provide important knowledge to the fields of fish, n-3 LCPUFA, and cognitive function in childhood, where the overall evidence is inconclusive. The behavioral, emotional, and stress response measurements will provide novel human results that may contribute to filling knowledge gaps on ways to increase mental robustness and prevent mental disorders starting from childhood.

The strengths of the study include the randomized controlled design with fasting blood samples and careful characterization of the study population, including measurements of dietary intake, objectively measured physical activity, and genotyping, as well as an objectively measured biomarker of compliance. This intense protocol and frequent consumption of study foods requires willingness and dedication of the participating families, and therefore the expected response rate will be only 1–4 %. Thus, the study population will expectedly not be fully representative of Danish children; however, the biological effects of consuming oily fish are not expected to differ between the study population and Danish children in general. The aim of 300 g/week of fish consumption is within the national recommendation [5], and this amount of oily fish is expected to provide approximately 0.8–1.0 g/day n-3 LCPUFA, a dose that has been shown to reduce blood pressure and plasma triacylglycerol in previous fish oil trials [12, 14, 15]. Despite our efforts to support children’s consumption of study foods by providing bone-free fish as well as poultry free of charge to the whole family, supply commonly eaten lunch products, and provide the parents with cooking tips and easy recipes, we expect that some children will not be able to reach an intake of 300 g/week. However, compared with previous fish oil trials in children, we still expect to provide a substantial amount of n-3 LCPUFA to the children while balancing what is achievable in a normal diet. Furthermore, this expected variation in children’s actual consumption of oily fish will allow us to explore potential dose-response effects of the fish on the many outcomes by use of n-3 LCPUFA in erythrocytes as a measure of oily fish intake during the intervention. Poultry has been chosen as the control food because it is the healthiest alternative and has comparable contents of protein and less n-3 LCPUFA than oily fish.

## Conclusions

The FiSK Junior study will help answer the question “What happens when children eat oily fish?” rather than “How can we make children eat oily fish?” The study will provide novel evidence to be used when setting dietary recommendations for children and thereby help optimize public health starting from childhood.

### Trial status

Recruitment was initiated in August 2016, and the last endpoint examination will be conducted in June 2017.

## Additional file

Additional file 1: Standard Protocol Items: Recommendations for Interventional Trials (SPIRIT) 2013 checklist. (PDF 57 kb)

## Abbreviations

BDNF: Brain-derived neurotrophic factor
CANTAB: Cambridge Neuropsychological Test Automated Battery
DDPCB: Dioxins and dioxin-like polychlorinated biphenyls
DHA: Docosahexaenoic acid (22:6n-3)
ECG: Electrocardiogram
FiSK: Fish, children, health, and cognition
HR: Heart rate
IGF: Insulin-like growth factor
LCPUFA: Long-chain polyunsaturated fatty acids
NEXS: Department of Nutrition, Exercise and Sports, University of Copenhagen
SPIRIT: Standard Protocol Items: Recommendations for Interventional Trials
TEQ: Toxic equivalents
TWI: Tolerable weekly intake
